# Identification of a Sex-Linked SNP Marker in the Salmon Louse (*Lepeophtheirus salmonis*) Using RAD Sequencing

**DOI:** 10.1371/journal.pone.0077832

**Published:** 2013-10-16

**Authors:** Stephen N. Carmichael, Michaël Bekaert, John B. Taggart, Hayden R. L. Christie, David I. Bassett, James E. Bron, Philip J. Skuce, Karim Gharbi, Rasmus Skern-Mauritzen, Armin Sturm

**Affiliations:** 1 Institute of Aquaculture, University of Stirling, Stirling, United Kingdom; 2 Moredun Research Institute, Penicuik, Midlothian, United Kingdom; 3 The GenePool, School of Biological Sciences, University of Edinburgh, Edinburgh, United Kingdom; 4 Marine Environmental Research Laboratory, University of Stirling, Machrihanish, Argyll, United Kingdom; 5 Institute of Marine Research, Bergen, Norway; University of Innsbruck, Austria

## Abstract

The salmon louse (*Lepeophtheirus salmonis* (Krøyer, 1837)) is a parasitic copepod that can, if untreated, cause considerable damage to Atlantic salmon (*Salmo salar* Linnaeus, 1758) and incurs significant costs to the Atlantic salmon mariculture industry. Salmon lice are gonochoristic and normally show sex ratios close to 1:1. While this observation suggests that sex determination in salmon lice is genetic, with only minor environmental influences, the mechanism of sex determination in the salmon louse is unknown. This paper describes the identification of a sex-linked Single Nucleotide Polymorphism (SNP) marker, providing the first evidence for a genetic mechanism of sex determination in the salmon louse. Restriction site-associated DNA sequencing (RAD-seq) was used to isolate SNP markers in a laboratory-maintained salmon louse strain. A total of 85 million raw Illumina 100 base paired-end reads produced 281,838 unique RAD-tags across 24 unrelated individuals. RAD marker *Lsa*101901 showed complete association with phenotypic sex for all individuals analysed, being heterozygous in females and homozygous in males. Using an allele-specific PCR assay for genotyping, this SNP association pattern was further confirmed for three unrelated salmon louse strains, displaying complete association with phenotypic sex in a total of 96 genotyped individuals. The marker *Lsa*101901 was located in the coding region of the prohibitin-2 gene, which showed a sex-dependent differential expression, with mRNA levels determined by RT-qPCR about 1.8-fold higher in adult female than adult male salmon lice. This study’s observations of a novel sex-linked SNP marker are consistent with sex determination in the salmon louse being genetic and following a female heterozygous system. Marker *Lsa*101901 provides a tool to determine the genetic sex of salmon lice, and could be useful in the development of control strategies.

## Introduction

Animals reproducing sexually can be divided into hermaphroditic species, in which at least some individuals are capable of producing both male and female gametes, and gonochoristic species, in which individuals are either male or female. In gonochoristic species, gender is the result of genetic and/or environmental sex determination. Depending on the species, sex determination may be controlled by either of these mechanisms or interactions of the two. In eutherian mammals, sex determination is genetic and defined by the male heterogametic XX/XY system [1]. The XX/XY system is also found in many invertebrates and has been suggested as the ancestral mechanism of genetic sex determination [1]. A female heterogametic system (ZW/ZZ) of genetic sex determination is evident in birds [2], some species of teleost fish [3], as well as several invertebrates including schistosomes [4] and lepidopteran insects [5]. In addition, several other genetic sex determination mechanisms have been described in insects, including the X-autosome balance system (XX/X0), diverse XX/XY systems involving several Y chromosomes, and the haploid/diploid system, in which males are haploid and females are diploid [6].

With about 67,000 characterised species, crustaceans represent a large and diverse group of invertebrates. Crustaceans are ecologically and economically important, as they provide keystone species in many ecosystems, including important aquaculture and wild fishery species, with 62 crustacean species accounting for 9.6% of global aquaculture production of food fish in 2010 [7]. Moreover, some crustaceans are damaging parasites of farmed fish and shellfish, while others have been reported as invasive species [8]. Given the rich diversity of crustacean species, it is perhaps not surprising that a wide variety of reproductive strategies exist in this group [9]. While hermaphroditism and parthenogenesis occur in different crustacean classes, most crustaceans are gonochoristic [10], with both environmental and genetic sex determination having been reported in the taxon. In addition, parasitic factors causing feminisation exist in different crustacean classes [11,12]. Early cytogenetic studies have provided evidence for a variety of male and female heterogametic systems in different crustacean taxa (reviewed in: Legrand et al) [9]. In penaeid shrimp (Decapoda) there is no evidence for environmental sex determination [13], and results from genetic mapping studies are in accordance with a female heterogametic (ZW/ZZ) system [14,15]. The ZW/ZZ system has further been found in *Macrobrachium rosenbergii* (giant freshwater prawn, Decapoda) [16] and *Armadillidium vulgare* (woodlouse, Isopoda) [17].

The salmon louse, *Lepeophtheirus salmonis* (Krøyer, 1837), is an ectoparasitic siphonostomatoid copepod of the family Caligidae that occurs on marine salmonids and has emerged as a major problem in mariculture of Atlantic salmon, *Salmo salar* Linnaeus, 1758 in the Northern hemisphere. Global Atlantic salmon production was estimated at 1.4 million tonnes in 2010 with a value of US $7.8 billion [18], while the cost of sea lice control was estimated in 2006 at US $480 million [19]. A small number of effective antiparasitics are now available for the treatment of *L. salmonis*, with reports of variable degrees of resistance to most medicinal agents currently licensed for use in Atlantic salmon production [20-22]. This has prompted increased research into *L. salmonis* biology and the molecular mechanisms of resistance, to explore potential non-chemical based *L. salmonis* control methods that can be utilised as part of an Integrated Pest Management (IPM) strategy [23]. Improved knowledge of the mechanisms controlling sex determination may further our understanding of how this process could be manipulated to contribute to sea louse control. Sex ratios observed in wild or laboratory populations of mobile *L. salmonis* are influenced by environmental factors and farm management practices but are usually close to 1:1 [24-27], which is consistent with a genetic mechanism of sex determination. However, conclusive data on the nature and mechanism of sex determination in this species are currently lacking.

The aim of the present study was to test the hypothesis that sex determination in *L. salmonis* is genetic, through the identification of sex-linked genetic markers. To this end, DNA from adult male and female *L. salmonis* was subjected to restriction site-associated DNA (RAD) sequencing, a powerful technique allowing simultaneous discovery and genotyping of Single Nucleotide Polymorphisms (SNPs) [28]. With on-going progress in unravelling the *L. salmonis* genome by the salmon louse genome project [http://sealouse.imr.no/], it is hoped that the results of this study will contribute to advancing our current understanding of sex determination and sex differentiation in *L. salmonis*.

## Materials and Methods

### Salmon louse strains

An *L. salmonis* laboratory-bred strain (S) that has previously been shown to be susceptible to all currently available anti-sea louse drugs [29] was used for RAD library preparation. Verification of SNP sex-association was performed using individuals from this strain and a further two unrelated laboratory-bred strains; an emamectin benzoate-resistant (PT) [29] and a strain recently established from a farm isolate (FI). These strains were founded from multiple individuals and have all been cultured under identical laboratory conditions, as described in detail elsewhere [29]. In brief, *L. salmonis* were maintained on Atlantic salmon with an initial weight of 500-1000 g in circular tanks supplied with fresh seawater at ambient temperature and salinity, using a photoperiod corresponding to natural day length. To propagate *L. salmonis*, egg strings were allowed to hatch and develop to copepodids, which were then used to inoculate a new tank of host fish. Prior to the collection of *L. salmonis* from hosts, fish were anaesthetised with 100 mg L^-1^ 2-phenoxyethanol. Infection rates were maintained at levels consistent with good fish welfare. All laboratory infections were carried out under UK Home Office licence and appropriate veterinary supervision [29].

### Salmon louse selection

*L. salmonis* engage in complicated courtship behaviour between adult males and late preadult II stage females, which culminates in the formation of pre-copula pairs [25]. Copulation takes place soon after the female moults into the adult stage, and females retain spermatophores from the mating in order to fertilise egg strings produced over their lifetime [25]. Adult male and preadult female (n = 24) *L. salmonis* from the S strain were used for RAD library preparation. Preadult females were selected in preference to adult females to avoid the possibility of sample contamination with stored sperm. Adult female salmon lice were used for the verification of SNP sex association in strain FI, after genital segments had been removed to avoid male DNA contamination. Adult male and preadult female *L. salmonis* are approximately the same size (total length ~5.4 mm) but can easily be distinguished at these stages of development under low magnification microscopy, using common morphological features [26]. The abdomen of adult male salmon lice is shorter than females with an ovoid genital complex, whereas the preadult II female genital complex is larger with cuticular folds and distinct lobes, and a narrowing of the abdomen as it meets the genital complex. Similarly, adult females have larger genital complexes than males and also have a larger more developed genital segment in comparison to preadult II females [26].

### RAD library preparation and sequencing

Adult male and preadult female *L. salmonis* (n = 24) from the S strain were collected from anaesthetised host fish as described above and allowed to recover for two hours in aerated filtered seawater at ambient sea temperature. The *L. salmonis* were then preserved in ethanol prior to storage at 4°C. Genomic DNA was extracted from individual *L. salmonis* using the REAL-Pure genomic DNA extraction kit (Durviz S.L., Spain), including removal of residual RNA through RNase A treatment of the extracts. UV spectroscopy (NanoDrop ND-1000, Thermo Scientific, USA) was used to confirm purity of the DNA samples and establish concentrations, whereas high molecular weight (MW) DNA integrity was assessed by agarose gel electrophoresis and ethidium bromide staining. Each high MW DNA sample was then diluted to a concentration of 45 ng/µl in 5 mM Tris, pH 8.5. The RAD libraries were prepared as detailed previously [30] with minor modifications as detailed in Houston et al [31]. Sequence details for the P1 and P2 paired-end adapters and library amplification primers used in RAD library preparation are available elsewhere [32]. Briefly, 200 ng of each DNA sample was digested at 37°C for 45 minutes with 2 units of *Pst*I high fidelity restriction enzyme (recognising the CTGC|AG motif) in a 10 µL reaction containing 1× Reaction Buffer 4 (New England Biolabs, UK). The reactions were then heat-inactivated at 80°C for 20 minutes. Each of the *Pst*I digested DNA samples were individually identified through the ligation of specific P1 adapters each containing a unique five base nucleotide barcode (Table S1), at 25°C for 30 minutes in a 12.5 µL reaction containing 100 nM P1 adapter, 200 units of T4 DNA Ligase, 1 mM rATP and 1× Reaction Buffer 2 (New England Biolabs, UK). Ligation reactions were heat inactivated at 65 °C for 20 minutes prior to combining them in four multiplexed libraries, each containing 12 salmon louse samples. Adaptive Focus Acoustics™ (AFA™) using the S220 High Performance Ultrasonicator (Covaris^®^ Inc., KBiosciences, UK.) was employed to randomly shear each RAD library pool to a size range of 150-700 bp. This sheared DNA was then column purified (PCR MinElute Kit, Qiagen) and size selected as previously described [31]. The RAD library construction protocol was then followed as published [28,30]. The RAD library pools were PCR amplified using 15-16 cycles and 150 µL of each amplified library was column purified, size selected (300-550 bp) and quality checked as previously described [31]. The four RAD library pools were further quality checked and quantified by qPCR (KAPA Library) prior to sequencing on one lane of the Illumina HiSeq 2000 platform (v3 chemistry) using 100 bp paired-end reads (EBI Sequence Read Archive (SRA) study ERP002400). Raw sequence data were processed using RTA 1.12.4.2 and Casava 1.6 (Illumina). RAD library qualitative and quantitative checks, Illumina sequencing and processing of raw sequence reads were performed at The GenePool Genomics Facility (University of Edinburgh, UK).

### Genotyping RAD alleles

Sequence reads with low quality scores (quality index score under 30, while the average quality score was 37), missing the restriction site or those with ambiguous barcodes (with more than one mismatch) were discarded from the sequence set. All the remaining sequence reads were then sorted into loci and genotyped, using the Stacks software 0.9995 [33]. The likelihood-based SNP calling algorithm [34] implemented in Stacks evaluated each nucleotide position for every RAD-tag from all individual samples, thereby differentiating true SNPs from sequencing errors. The processing parameters used in Stacks included; a minimum stack depth of at least 30 sequences, a maximum of 2 mismatches in each locus for each individual and up to 1 mismatch between alleles. The paired-end reads were assembled using both Stacks and Velvet (version 1.2.08) software [35], which were used to separate RAD-tag sequences, with or without potential SNPs, but belonging to separate candidate loci.

### SNP sex-association

The genetic association of *L. salmonis* phenotypic sex with RAD marker alleles was carried out by counting the number of times each allele was associated with a particular sex. These counts were compared to an ideal scenario where each allele would be specific to a sex.

### Verification of SNP sex-association

An additional twelve adult male and twelve preadult female *L. salmonis* per strain were sampled from strains S and PT and preserved in ethanol as detailed above. Similarly, twelve adult male and adult female *L. salmonis* were sampled from strain FI. Genomic DNA was extracted from each *L. salmonis* individual using the REAL-Pure genomic DNA extraction kit (Durviz S.L., Spain), quality checked and diluted as detailed above (45 ng/µL in 5 mM Tris, pH 8.5). SNP marker sex-association was verified using an allele specific PCR genotyping assay (KASP^TM^ v4.0, LGC Genomics, UK). SNP-specific primers were designed by LGC Genomics using sequence flanking RAD-marker *Lsa*101901 (Table S2). For each of the three strains, twelve male and twelve female samples were genotyped in duplicate 10 µL reactions each containing approximately 40 ng template DNA, using the following amplification conditions: 94°C for 15 minutes followed by 35 cycles of 94°C for 20 seconds then touch-down cycles over 61-55°C for 60 seconds (dropping 0.6°C per cycle). Individual *L. salmonis* genotype assignment was performed through reading the fluorescence emission of the FAM and CAL Fluor Orange 560 fluorophores for each sample, in comparison to no-template control reactions, using endpoint genotyping software and the Quantica qPCR thermal cycler (Bibby Scientific, UK).

### RT-qPCR analysis of prohibitin-2 expression

The mRNA abundance of the prohibitin-2 gene was determined in adult male (n = 10) and female (n = 8) drug susceptible (S) *L. salmonis* by reverse transcription quantitative PCR (RT-qPCR), using relative quantification with two reference genes that had shown stable expression levels in previous experiments (Hypoxanthine-guanine phosphoribosyltransferase (HGPRT) and Required for meiotic nuclear division 5 (RMD-5) homolog) (unpublished data). Primers were designed for these three genes with melting temperatures (T_m_) of ~60°C using Primer 3 software (Table S3). Adult male and female salmon lice were collected from anaesthetised host fish as described above and allowed to recover for 2 hours in aerated filtered seawater at ambient sea temperature and then preserved in an RNA stabilisation solution (4.54M ammonium sulphate, 25mM trisodium citrate, 20mM EDTA, pH 5.4) prior to storage at -70°C. Individual frozen salmon lice were ground in liquid nitrogen using a pestle and mortar, and total RNA was immediately extracted from the homogenised sample using TRI Reagent^®^ (Sigma-Aldrich, UK), following the manufacturers’ protocol. After phase separation, RNA was precipitated from the aqueous phase by addition of 0.25 volumes isopropanol and 0.25 volumes of a high salt buffer (0.8 M trisodium citrate; 1.2 M sodium chloride), as recommended for samples with high polysaccharide content [36]. The total RNA was resuspended in nuclease-free water. UV spectroscopy (NanoDrop ND-1000, Thermo Scientific, USA) was used to confirm purity of the RNA samples and establish concentrations, whereas RNA quality was assessed by agarose gel electrophoresis and ethidium bromide staining. Aliquots (1 µg) of total RNA from adult male or female *L. salmonis* were reverse transcribed (Superscript III, Invitrogen, UK) using random hexamers and anchored oligo-dT in a 3:1 molar ratio. No-template controls and controls omitting RT enzyme were included on each assay plate to detect potential DNA contamination. A cDNA pool containing equal amounts of all samples was made and included on each assay plate at different dilutions to allow the derivation of a standard curve. RT-qPCR reactions were performed in duplicate in a total volume of 20 µL containing 5 µL sample cDNA (20-fold dilution), 0.3 µM of each primer and 10 µL Absolute SYBR Green I mix (ThermoFisher Scientific, UK), using the Mastercycler ep realplex^2^ (Eppendorf, UK) with the following amplification conditions: 95°C for 15 minutes, followed by 40 cycles of 94°C for 30 seconds, 55°C for 15 seconds and 72°C for 30 seconds. After amplification a melt curve from 55°C to 95°C at 0.5°C increments for 15 seconds each was performed to ensure that a single product was amplified in each reaction. Threshold cycles were analysed using the PCR cycler software. Standard curves were derived from plots of the threshold cycle against the logarithm of the relative concentration of cDNA pool. Primer efficiency (E) was derived from linear fits to the standard curve according to the equation E = 10^(-1/slope)^. The BestKeeper tool [37] was employed to analyse expression stability of two reference genes and determine a robust BestKeeper expression index as a geometric mean for the two reference genes, which was in turn used to establish relative gene expression ratios using the ΔΔCt method (Ratio = (E_target_) ^ΔCt target (control – sample)^ / (E_reference_) ^ΔCt reference (control – sample)^) in the Relative Expression Software Tool (REST) Multiple Condition Solver (MCS) software [38].

Relative expression ratios from RT-qPCR analysis were compared between male and female *L. salmonis* using the non-parametric Mann-Whitney test as implemented in the Minitab 16.1 software package (Minitab Inc., UK). The significance level was set at p<0.05.

### Ethics statement

All experimental research reported in this study was performed in accordance with the U.K. Home Office regulations regarding the use of animals in experiments and testing (Project license: 60/3848) and was approved by the University of Stirling Research Ethics Committee.

## Results

### RAD sequencing

**Figure 1:**
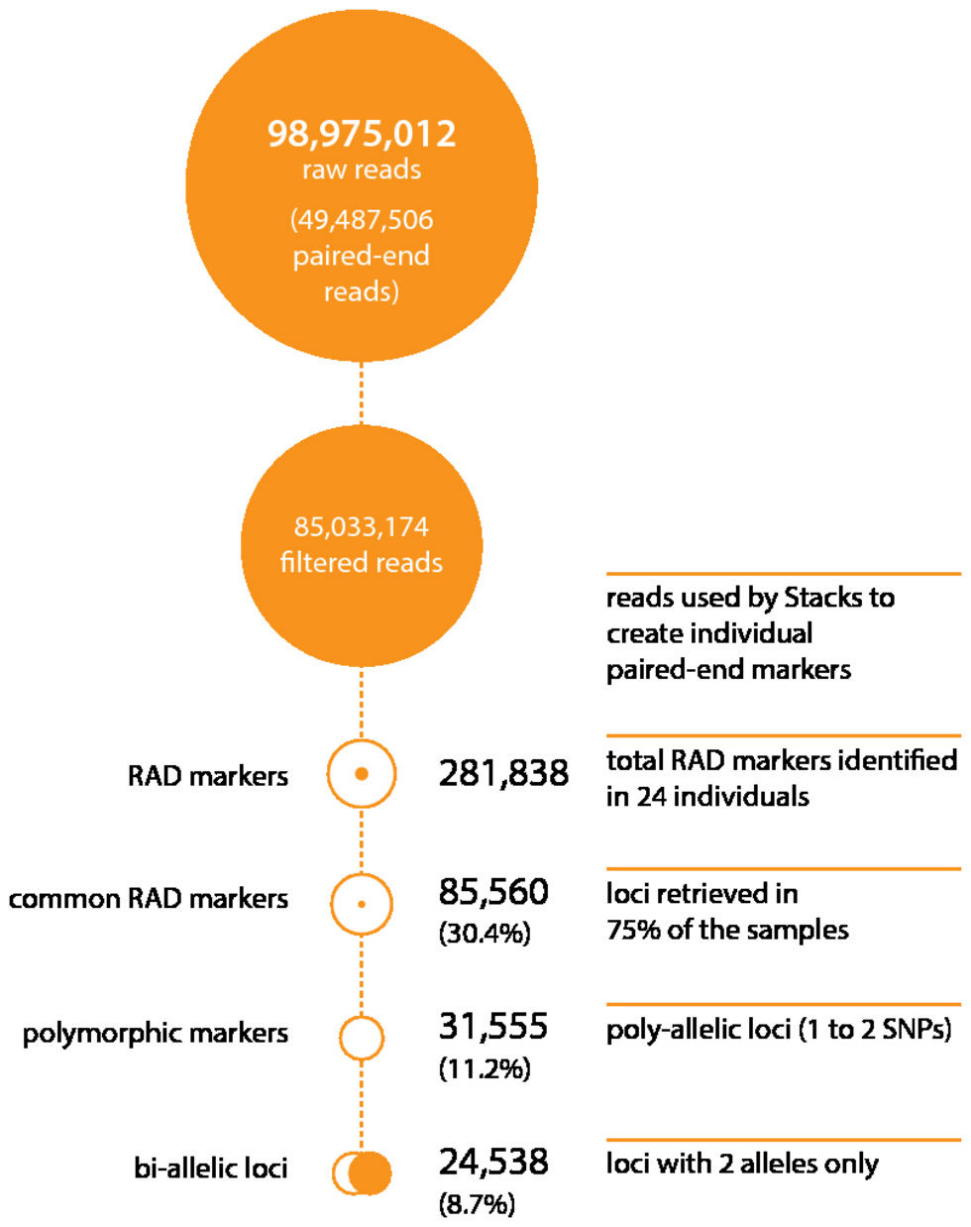
*L. salmonis* RAD sequencing and polymorphic marker identification. RAD-seq technology was employed to genotype 24 individuals from a drug susceptible (S) laboratory-maintained *L. salmonis* strain. This produced 98,975,012 raw sequence reads that comprised 49,487,506 paired-end reads although after filtering 85,033,174 raw sequence reads remained that produced 281,838 paired-end RAD markers. 31,555 of these polymorphic markers were poly-allelic and present in at least 75% of the individuals analysed.

DNA from each of 12 male and 12 female individuals from the drug susceptible (S) laboratory-maintained *L. salmonis* strain [29] was used to generate multiplexed *Pst*I RAD libraries and sequenced at high depth using the Illumina HiSeq 2000 platform. In total, 98,975,012 raw reads (100 nt long) were produced, that comprised 49,487,506 paired-end reads (EBI Sequence Read Archive (SRA) study ERP002400). After removal of low quality sequence reads (quality index score under 30), sequences with ambiguous barcodes and orphaned paired-end reads, 85.9% of the raw reads were retained (85,033,174 reads). The Stacks package [33] was then used to assemble loci (RAD-tags) for each individual, which produced 281,838 unique RAD-tags (Figure 1). The raw sequence read count and RAD-tag count for each sample are reported in Table S1.

### SNP sex-association

Initial analysis of read number for the 281,838 RAD-tags did not reveal any sex-specific markers (i.e. present in only one of the sexes). To maximise the number of informative markers and minimise the amount of missing or erroneous data, we then used only paired-end RAD-tags retrieved from at least 75% of the samples in each gender group, which resulted in the retention of 85,560 RAD-tags (Figure 1). Analysis of this filtered set of markers did not reveal any RAD-tags with twice the coverage in one gender compared to the other. Further analysis revealed that 31,555 of these RAD-tags were polymorphic (containing 1 or 2 SNPs), of which 24,538 were bi-allelic (Figure 1). The genetic association of polymorphic markers with sex was performed by direct comparison of each allele with the phenotypic sex of the individual. The results were then ranked in order, from maximum (complete separation between male and female) to minimum association (not significantly different from random association). Evaluation of the 24,538 bi-allelic markers identified only one marker that exhibited complete association with gender, with all samples having a heterozygous female (allele ‘G’ and ‘T’) or homozygous male (allele ‘G’ only) genotype (*Lsa*101901; NCBI dbSNP Accession: 749737482; Table S2). The mean read number at this locus was 29 reads; female heterozygous alleles showed a mean read number of 14.5 each, whereas the male homozygous allele had 29 reads.

### Verification of sex association

**Figure 2:**
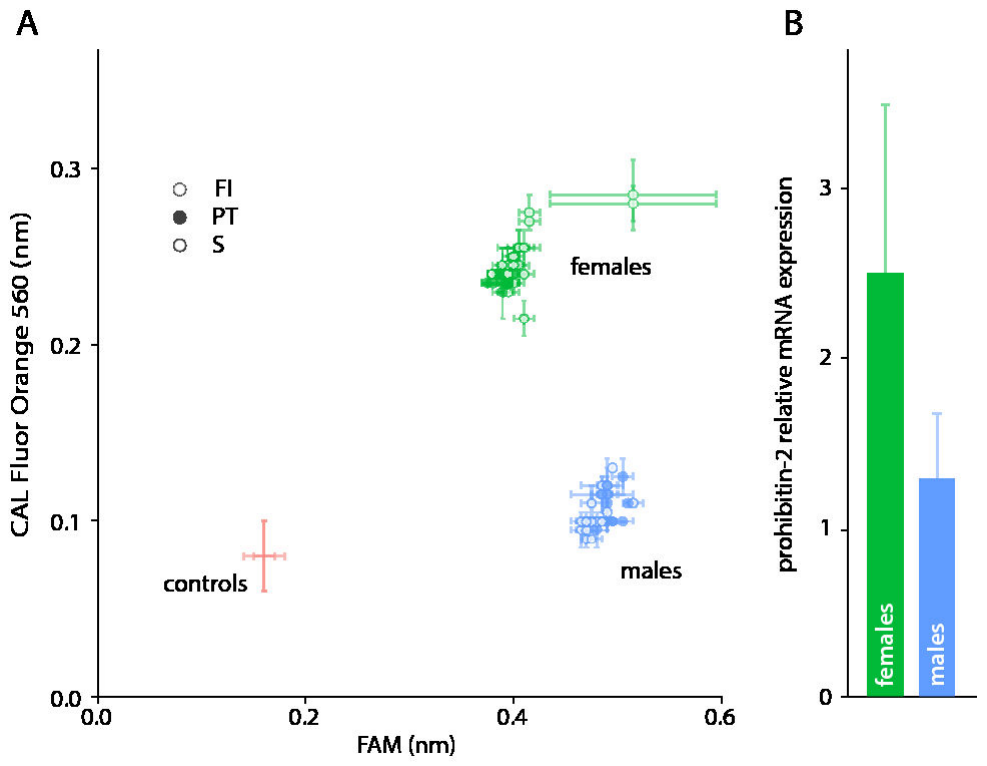
Analysis of prohibitin-2 in male and female *L. salmonis*. (**A**) Genotyping using the allele specific KASP assay. A total of 72 individuals (36 male and 36 female) from three unrelated *L. salmonis* strains (S, PT and FI) were genotyped using an allele specific PCR assay (KASP^TM^, LGC Genomics, UK). Individual *L. salmonis* genotype assignment was performed through reading the fluorescence emission of the FAM (Allele 1) and CAL Fluor Orange 560 (Allele 2) fluorophores for each sample, in comparison to no-template control reactions. The results of this PCR genotyping assay confirmed complete association of SNP genotype with *L. salmonis* sex as identified by RAD-seq analysis. (**B**) Differential expression of prohibitin-2. Relative prohibitin-2 expression (AVG ± SD) is shown for adult female (n = 8) and adult male (n = 10) *L. salmonis* from the drug susceptible (S) laboratory-maintained strain. The prohibitin-2 expression differed significantly between *L. salmonis* males and females (p = 0.0117, Mann-Whitney test).

The association of marker *Lsa*101901 to phenotypic sex was further investigated using an allele specific PCR genotyping assay (KASP^TM^, LGC Genomics, UK). Individuals genotyped for the marker first included 12 male and 12 female lice from strain S that were unrelated to the *L. salmonis* used to generate the RAD library. Twelve males and 12 females from each of two further laboratory maintained *L. salmonis* strains PT and FI were also analysed. In all tested individuals, a complete association of the marker with phenotypic sex was observed, with females being heterozygous (G/T) and males homozygous (G/G) (Figure 2A).

### SNP marker localisation

The 218 bp marker sequence containing the *Lsa*101901 sex-linked SNP was used as a query in a nucleotide BLAST (Basic Local Alignment Search Tool) search against the non-redundant nucleotides (nr) database available in GenBank at the National Centre for Biotechnology Information (NCBI). The marker sequence containing SNP *Lsa*101901 was identical to *L. salmonis* putative prohibitin-2 sequence (Accession BT121810.1, BLASTn e-value 2×10^-109^). The SNP in marker *Lsa*101901 was found to be a synonymous polymorphism within the coding region of the prohibitin-2 gene. A BLASTx search against the NCBI Reference Proteins (refseq_protein) database further established the identity of the marker-containing sequence, as it showed a high similarity to a prohibitin-2-like protein (Accession XP003746427.1) from *Metaseiulus occidentalis* (Western predatory mite): 88% identity across the whole sequence (BLASTx e-value 2×10^-37^). The two *Lsa*101901 marker allele sequences were also identified in EST sequences (100% query coverage) from Canadian and Norwegian Atlantic *L. salmonis* populations in addition to the Pacific population, using a BLASTn search against the NCBI EST database (Table 1).

**Table 1:** Identification of *L. salmonis* EST sequences representing marker *Lsa*101901 alleles.

*L. salmonis* population	Allele	EST Accession
Atlantic Norway	G	GW663052.1
Atlantic Norway	T	HO677162.1
Atlantic Canada	G	GW644163.1
Atlantic Canada	T	GW642628.1, GW642629.1
Pacific	G	FK914464.1, EX486009.1
Pacific	T	FK913245.1, FK913246.1

### Gene expression analysis of prohibitin-2

RT-qPCR analysis demonstrated that the marker sequence, containing the SNP *Lsa*101901 and annotated as *L. salmonis* prohibitin-2, was significantly differentially expressed (p = 0.0117, Mann-Whitney test) between male (n = 10) and female (n = 8) *L. salmonis* from a drug susceptible laboratory-maintained strain (Figure 2B). Relative expression analysis found that adult female *L. salmonis* expressed 1.8 fold more prohibitin-2 mRNA compared to adult males from this strain.

## Discussion

In the present study a SNP marker has been identified in *L. salmonis* that showed complete association with sex in 96 genotyped individuals from three different strains. The results strongly suggest that sex determination in *L. salmonis* is genetic, and provide evidence for a female heterogametic ZW/ZZ system. Effects of environmental factors on sex determination have been described for a number of free-living and parasitic copepods [39,40]. However, sex ratios close to 1:1 have been observed in laboratory studies with *L. salmonis* [26,27], which is in accordance with this study’s suggestion of a genetic sex determination mechanism.

Cytogenetic investigations and studies of sex-linked marker heritability have suggested diverse systems of genetic sex determination in crustaceans, with the most common ones being based on male (XX/XY) or female heterogamety (ZW/ZZ) (reviewed in: Legrand et al) [9]. In decapods, genetic linkage maps have provided evidence for ZW/ZZ systems in a number of penaeid shrimps and a freshwater prawn [14,15,41], whereas cytogenetic studies have suggested male heterogametic systems (XX/XY or X0/XX) in brachyuran crabs (reviewed in: Lecher et al) [42]. Cytogenetic data further provide evidence for the presence of both male and female heterogametic sex determination systems among Copepoda (reviewed in: Legrand et al) [9]. The available data thus illustrate that mechanisms of sex determination are not conserved among crustaceans, which parallels the situation in insects [6]. Interestingly, the divergent sex determination systems of insects share an evolutionarily conserved pathway involving the *transformer* gene and its downstream target *doublesex*, but differ with respect to an upstream switching mechanism [6]. Homologues to the sex determination-related insect genes *fruitless*, *sex lethal* and *transformer* [43] have been reported from *Penaeus monodon* (giant tiger prawn) [44] and *Macrobrachium nipponense* (oriental river shrimp) [45]. Moreover, a homologue of *doublesex* has been shown to be involved in environmental sex determination in the branchiopod *Daphnia magna* [46]. Together, this suggests that molecular pathways of sex determination are partially conserved between insects and crustaceans.

Relatively little is known concerning sex differentiation and its endocrine control in crustaceans, and most available data have been obtained on decapods (reviewed in: Rodriguez et al) [47]. In this group, the default route of sexual development is female. Male sexual differentiation requires the presence of a male endocrine organ called the androgenic gland that produces an insulin-like factor controlling testis function [41]. Activity of the androgenic gland in males and ovaries in females is negatively controlled by the gonad-inhibiting hormone (GIH) and positively controlled by the gonad-stimulating hormone (GSH) [47]. Further hormones with roles in crustacean reproduction include methyl farnesoate, which is homologous to the juvenile hormones controlling metamorphosis in insects. Methyl farnesoate stimulates ovarian growth in decapods [48], and induces the production of male offspring in cladocerans [49]. Ecdysteroids are insect and crustacean hormones regulating the moulting process (ecdysis), and have been shown to stimulate ovarian growth in some crustaceans [46]. Exposure to the ecdysteroid 20-hydroxyecdysone increased the number of male offspring in the branchiopod *Daphnia pulex* (Water flea) and the copepod *Tisbe battagliai* [50,51]. Some studies have further suggested roles for steroids in crustacean reproduction; however, the precise identity and function of steroid hormones in crustaceans is still unknown [47,52].

In the present study, a sex-linked SNP marker was isolated in *L. salmonis* and was shown to correspond to a synonymous polymorphism in a gene encoding a homologue to prohibitin-2. Prohibitin-2 and the related prohibitin-1 are highly conserved ubiquitous eukaryotic proteins found in the mitochondria, where they have been suggested to function as chaperone proteins [53]. Prohibitins are also found in the nucleus, where they may regulate gene expression through interaction with a wide variety of transcription factors including steroid receptors. Prohibitin-2, also known as repressor of oestrogen receptor activity (REA), binds directly to the oestrogen receptor (ER), acting as a negative co-regulator of transcriptional activity [54]. In targeted gene disruption studies with mice, the homozygous null mutation of prohibitin-2 was lethal, whereas in heterozygous knockouts increased physiological responses to oestrogens were observed in females, but not males [55]. Gene disruption studies in the nematode *Caenorhabditis elegans* further provide evidence for roles of prohibitin-1 in gametogenesis [56]. A study of a prohibitin homologue in *Eriocheir sinensis* (Chinese mitten crab) suggested a role in spermatogenesis [57], whereas an investigation in *P. monodon* demonstrated prohibitin-2 mRNA expression in both male and female gonads [44]. In summary, published studies provide some evidence for sex-specific roles of prohibitins, which is in accordance with this study’s finding of significantly higher mRNA levels of a prohibitin-2 homologue in adult female when compared to adult male *L. salmonis*. However, no evidence exists for a role for prohibitins in sex determination and/or sex differentiation. Following from these findings, it is clear that further research will be required to elucidate the nature of the molecular determinant(s) of sex determination in *L. salmonis* and to clarify the relationships that such determinants may have to the SNP marker reported in this study.

## Conclusions

A novel sex-linked SNP marker showing complete association with sex has been identified in the salmon louse. The data suggest a genetic mechanism of sex determination in *L. salmonis* based on female heterozygosity. The SNP marker represents a synonymous polymorphism in a prohibitin-2 homologue, however, the functional relationship of prohibitin-2 to sex determination remains uncertain. These findings contribute towards an improved understanding of sex determination in sea lice and may serve to help develop improved control strategies for this species.

## Supporting Information

Table S1 **Multiplex barcode assignment and RAD-tag identification for individual samples.** Unique five base nucleotide barcodes were assigned to each *L. salmonis* DNA sample. These samples were included in a multiplex RAD library and sequenced, which generated sequence reads that were quality filtered and used for the identification of paired-end RAD-tags in at least 75% of the samples.
(XLSX)

Table S2 ***L. salmonis* SNP marker and KASP assay primer sequences.** Two SNP alleles and RAD-tag allele sequences that were identified as the SNP marker *Lsa101901* are detailed, along with the allele specific primers and common primer designed for the allele specific PCR genotyping assay (KASP^TM^, LGC Genomics, UK).
(XLSX)

Table S3 **Primers used for RT-qPCR analysis of prohibitin-2 expression in male and female adult *L. salmonis*.**
(XLSX)

## References

[1] Charlesworth D, Mank JE (2010) The birds and the bees and the flowers and the trees: Lessons from genetic mapping of sex determination in plants and animals. Genetics 186: 9-31. doi:10.1534/genetics.110.117697. PubMed: 20855574. 20855574 PMC2940314

[2] Nakamura M (2010) The mechanism of sex determination in vertebrates-are sex steroids the key-factor? J Exp Zool A Ecol Genet Physiol 313: 381-398. PubMed: 20623803. 20623803 10.1002/jez.616

[3] Cnaani A, Lee BY, Zilberman N, Ozouf-Costaz C, Hulata G et al. (2008) Genetics of sex determination in tilapiine species. Sex Dev 2: 43-54. doi:10.1159/000117718. PubMed: 18418034. 18418034

[4] Criscione CD, Valentim CLL, Hirai H, LoVerde PT, Anderson TJC (2009) Genomic linkage map of the human blood fluke *Schistosoma* *mansoni* . Genome Biol 10: R71. doi:10.1186/gb-2009-10-6-r71. PubMed: 19566921. 19566921 PMC2718505

[5] Ohbayashi F, Suzuki MG, Shimada T (2002) Sex determination in *Bombyx* *mori* . Curr Sci 83: 466-471.

[6] Gempe T, Beye M (2011) Function and evolution of sex determination mechanisms, genes and pathways in insects. BioEssays 33: 52-60. doi:10.1002/bies.201000043. PubMed: 21110346. 21110346 PMC3040292

[7] FAO (2012a) The state of World Fisheries and Aquaculture 2012. Rome. 209 p. Available: http://www.fao.org/docrep/016/i2727e/i2727e00.htm. [Accessed 18 March 2013].

[8] Hänfling B, Edwards F, Gherardi F (2011) Invasive alien Crustacea: Dispersal, establishment, impact and control. BioControl 56: 573-595. doi:10.1007/s10526-011-9380-8.

[9] Legrand JJ, Legrandhamelin E, Juchault P (1987) Sex Determination in Crustacea. Biol Rev Camb Philos Soc 62: 439-470. doi:10.1111/j.1469-185X.1987.tb01637.x.

[10] Juchault P (1999) Hermaphroditism and gonochorism. A new hypothesis on the evolution of sexuality in Crustacea. C R Acad Sci III 322: 423-427. doi:10.1016/S0764-4469(99)80078-X.

[11] Bouchon D, Rigaud T, Juchault P (1998) Evidence for widespread *Wolbachia* infection in isopod crustaceans: Molecular identification and host feminization. Proc R Soc Edinb Biol 265 pp. 1081-1090. PubMed: 9684374. 10.1098/rspb.1998.0402 PMC1689171 9684374

[12] Voordouw MJ, Stebbins G, Robinson HE, Perrot-Minnot MJ, Rigaud T et al. (2008) Genetic variation in the primary sex ratio in populations of the intertidal copepod, *Tigriopus* *californicus*, is widespread on Vancouver Island. Evol Ecol Res 10: 1007-1023.

[13] Campos-Ramos R, Garza-Torres R, Guerrero-Tortolero DA, Maeda-Martínez AM, Obregón-Barboza H (2006) Environmental sex determination, external sex differentiation and structure of the androgenic gland in the Pacific white shrimp *Litopenaeus* *vannamei* (Boone). Aquacult Res 37: 1583-1593. doi:10.1111/j.1365-2109.2006.01604.x.

[14] Li Y, Byrne K, Miggiano E, Whan V, Moore S et al. (2003) Genetic mapping of the kuruma prawn *Penaeus* *japonicus* using AFLP markers. Aquaculture 219: 143-156. doi:10.1016/S0044-8486(02)00355-1.

[15] Staelens J, Rombaut D, Vercauteren I, Argue B, Benzie J et al. (2008) High-density linkage maps and sex-linked markers for the black tiger shrimp (*Penaeus* *monodon*). Genetics 179: 917-925. doi:10.1534/genetics.107.080150. PubMed: 18558652. 18558652 PMC2429885

[16] Malecha SR, Nevin PA, Ha P, Barck LE, Lamadrid-Rose Y et al. (1992) Sex-ratios and sex-determination in progeny from crosses of surgically sex-reversed freshwater prawns, *Macrobrachium* *rosenbergii* . Aquaculture 105: 201-218. doi:10.1016/0044-8486(92)90087-2.

[17] Juchault P, Legrand JJ (1972) Crossing of experimental neo-males in *Armadillidium* *vulgare* Latr - (Crustacea, Isopoda, Oniscoidea) - Presentation of female heterogamy. C R Acad Sci Hebd Seances Acad Sci D. p. 274: 1387 4622077

[18] FAO (2012b) FAO Yearbook - Fishery and Aquaculture Statistics 2010. Rome. 239 p. Available: http://www.fao.org/fishery/publications/2012/en. [Accessed 18 March 2013].

[19] Costello MJ (2009) The global economic cost of sea lice to the salmonid farming industry. J Fish Dis 32: 115-118. doi:10.1111/j.1365-2761.2008.01011.x. PubMed: 19245636. 19245636

[20] Denholm I, Devine GJ, Horsberg TE, Sevatdal S, Fallang A et al. (2002) Analysis and management of resistance to chemotherapeutants in salmon lice, *Lepeophtheirus* *salmonis* (Copepoda : Caligidae). Pest Manag Sci 58: 528-536. doi:10.1002/ps.482. PubMed: 12138619. 12138619

[21] Sevatdal S, Horsberg TE (2003) Determination of reduced sensitivity in sea lice (*Lepeophtheirus* *salmonis* Kroyer) against the pyrethroid deltamethrin using bioassays and probit modelling. Aquaculture 218: 21-31. doi:10.1016/S0044-8486(02)00339-3.

[22] Horsberg TE (2012) Avermectin use in aquaculture. Curr Pharm Biotechnol 13: 1095-1102. doi:10.2174/138920112800399158. PubMed: 22039799. 22039799

[23] Torrissen O, Jones S, Asche F, Guttormsen A, Skilbrei OT et al. (2013) Salmon lice - impact on wild salmonids and salmon aquaculture. J Fish Dis 36: 171-194. doi:10.1111/jfd.12061. PubMed: 23311858. 23311858 PMC3675643

[24] Bron JE, Sommerville C, Wooten R, Rae GH (1993) Influence of treatment with dichlorvos on the epidemiology of *Lepeophtheirus* *salmonis* (Krøyer 1837) and *Caligus* *elongates* Nordmann 1832 on Scottish salmon farms. In: Boxhall GA Defaye D Pathogens of wild and farmed fish: sea lice. London: Ellis Horwood Ltd. pp. 263 - 274.

[25] Ritchie G, Mordue AJ, Pike AW, Rae GH (1996) Observations on mating and reproductive behaviour of *Lepeophtheirus* *salmonis*, Kroyer (Copepoda: Caligidae). J Exp Mar Biol Ecol 201: 285-298. doi:10.1016/0022-0981(96)00008-1.

[26] Johnson SC, Albright LJ (1991) Development, growth, and survival of *Lepeophtheirus* *salmonis* (Copepoda, Caligidae) under laboratory conditions. J Mar Biol Assoc UK 71: 425-436. doi:10.1017/S0025315400051687.

[27] Hamre LA, Glover KA, Nilsen F (2009) Establishment and characterisation of salmon louse (*Lepeophtheirus* *salmonis* (Kroyer 1837)) laboratory strains. Parasitol Int 58: 451-460. doi:10.1016/j.parint.2009.08.009. PubMed: 19732850. 19732850

[28] Baird NA, Etter PD, Atwood TS, Currey MC, Shiver AL et al. (2008) Rapid SNP discovery and genetic mapping using sequenced RAD markers. PLOS ONE 3: e3376. doi:10.1371/journal.pone.0003376. PubMed: 18852878. 18852878 PMC2557064

[29] Heumann J, Carmichael S, Bron JE, Tildesley A, Sturm A (2012) Molecular cloning and characterisation of a novel P-glycoprotein in the salmon louse *Lepeophtheirus* *salmonis* . Comp Biochem Physiol C Pharmacol Toxicol Endocrinol 155: 198-205. doi:10.1016/j.cbpc.2011.08.004. PubMed: 21867772. 21867772

[30] Etter PD, Bassham S, Hohenlohe PA, Johnson EA, Cresko WA (2011) SNP discovery and genotyping for evolutionary genetics using RAD sequencing. Methods Mol Biol 772: 157-178. PubMed: 22065437. 22065437 10.1007/978-1-61779-228-1_9 PMC3658458

[31] Houston RD, Davey JW, Bishop SC, Lowe NR, Mota-Velasco JC et al. (2012) Characterisation of QTL-linked and genome-wide restriction site-associated DNA (RAD) markers in farmed Atlantic salmon. BMC Genomics 13: 244. doi:10.1186/1471-2164-13-244. PubMed: 22702806. 22702806 PMC3520118

[32] Baxter SW, Davey JW, Johnston JS, Shelton AM, Heckel DG et al. (2011) Linkage mapping and comparative genomics using next-generation RAD sequencing of a non-model organism. PLOS ONE 6: e19315. doi:10.1371/journal.pone.0019315. PubMed: 21541297. 21541297 PMC3082572

[33] Catchen JM, Amores A, Hohenlohe P, Cresko W, Postlethwait JH (2011) Stacks: Building and genotyping loci de novo from short-read sequences. G3 1: 171-182. PubMed: 22384329. 22384329 10.1534/g3.111.000240 PMC3276136

[34] Hohenlohe PA, Bassham S, Etter PD, Stiffler N, Johnson EA et al. (2010) Population genomics of parallel adaptation in threespine stickleback using sequenced RAD tags. PLOS Genet 6: e1000862 PubMed: 20195501. 20195501 10.1371/journal.pgen.1000862 PMC2829049

[35] Zerbino DR, Birney E (2008) Velvet: Algorithms for de novo short read assembly using de Bruijn graphs. Genome Res 18: 821-829. doi:10.1101/gr.074492.107. PubMed: 18349386. 18349386 PMC2336801

[36] Chomczynski P, Mackey K (1995) Modification of the TRI-reagent procedure for isolation of RNA from polysaccharide-rich and proteoglycan-rich sources. BioTechniques 19: 942 - 945. PubMed: 8747660. 8747660

[37] Pfaffl MW, Tichopad A, Prgomet C, Neuvians TP (2004) Determination of stable housekeeping genes, differentially regulated target genes and sample integrity: BestKeeper - Excel-based tool using pair-wise correlations. Biotechnol Lett 26: 509-515. doi:10.1023/B:BILE.0000019559.84305.47. PubMed: 15127793. 15127793

[38] Pfaffl MW (2001) A new mathematical model for relative quantification in real-time RT-PCR. Nucleic Acids Res 29: e45. doi:10.1093/nar/29.9.e45. PubMed: 11328886. 11328886 PMC55695

[39] Gusmão LFM, McKinnon AD (2009) Sex ratios, intersexuality and sex change in copepods. J Plankton Res 31: 1101-1117. doi:10.1093/plankt/fbp059.

[40] Michaud M, De Mee ȗ s T, Renaud F (2004) Environmental sex determination in a parasitic copepod: Checking heterogeneity and unpredictability of the environment. Mar Ecol Prog Ser 269: 163-171. doi:10.3354/meps269163.

[41] Ventura T, Aflalo ED, Weil S, Kashkush K, Sagi A (2011) Isolation and characterization of a female-specific DNA marker in the giant freshwater prawn *Macrobrachium* *rosenbergii* . Heredity 107: 456-461. doi:10.1038/hdy.2011.32. PubMed: 21522169. 21522169 PMC3199927

[42] Lecher P, Defaye D, Noel P (1995) Chromosomes and nuclear DNA of Crustacea. Int J Invert Reprod Dev 27: 85-114. doi:10.1080/07924259.1995.9672440.

[43] Kopp A (2012) Dmrt genes in the development and evolution of sexual dimorphism. Trends Genet 28: 175-184. doi:10.1016/j.tig.2012.02.002. PubMed: 22425532. 22425532 PMC3350790

[44] Leelatanawit R, Sittikankeaw K, Yocawibun P, Klinbunga S, Roytrakul S et al. (2009) Identification, characterization and expression of sex-related genes in testes of the giant tiger shrimp *Penaeus* *monodon* . Comp Biochem Physiol A Mol Integr Physiol 152: 66-76. doi:10.1016/j.cbpa.2008.09.004. PubMed: 18824117. 18824117

[45] Qiao H, Fu H, Jin S, Wu Y, Jiang S et al. (2012) Constructing and random sequencing analysis of normalized cDNA library of testis tissue from oriental river prawn (*Macrobrachium* *nipponense*). Comp Biochem Physiol D Genomics Proteomics 7: 268-276. doi:10.1016/j.cbd.2012.04.003. PubMed: 22632994. 22632994

[46] Kato Y, Kobayashi K, Watanabe H, Iguchi T (2011) Environmental sex determination in the branchiopod crustacean *Daphnia* *magna*: Deep conservation of a Doublesex gene in the sex-determining pathway. PLOS Genet 7: e1001345 PubMed: 21455482. 21455482 10.1371/journal.pgen.1001345 PMC3063754

[47] Rodríguez EM, Medesani DA, Fingerman M (2007) Endocrine disruption in crustaceans due to pollutants: A review. Comp Biochem Physiol A Mol Integr Physiol 146: 661-671. doi:10.1016/j.cbpa.2006.04.030. PubMed: 16753320. 16753320

[48] Laufer H, Biggers WJ, Ahl JSB (1998) Stimulation of ovarian maturation in the crayfish *Procambarus* *clarkii* by methyl farnesoate. Gen Comp Endocrinol 111: 113-118. doi:10.1006/gcen.1998.7109. PubMed: 9679083. 9679083

[49] Olmstead AW, LeBlanc GA (2003) Insecticidal juvenile hormone analogs stimulate the production of male offspring in the crustacean *Daphnia* *magna* . Environ Health Perspect 111: 919-924. doi:10.1289/ehp.5982. PubMed: 12782492. 12782492 PMC1241525

[50] Peterson JK, Kashian DR, Dodson SI (2001) Methoprene and 20-OH-ecdysone affect male production in *Daphnia* *pulex* . Environ Toxicol Chem 20: 582-588. doi:10.1002/etc.5620200318. PubMed: 11349860. 11349860

[51] Hutchinson TH, Pounds NA, Hampel M, Williams TD (1999) Life-cycle studies with marine copepods (*Tisbe* *battagliai*) exposed to 20-hydroxyecdysone and diethylstilbestrol. Environ Toxicol Chem 18: 2914-2920. doi:10.1002/etc.5620181237.

[52] Mazurová E, Hilscherová K, Triebskorn R, Köhler HR, Maršálek B et al. (2008) Endocrine regulation of the reproduction in crustaceans: Identification of potential targets for toxicants and environmental contaminants. Biologica 63: 139-150. doi:10.2478/s11756-008-0027-x.

[53] Mishra S, Murphy LC, Murphy LJ (2006) The prohibitins: Emerging roles in diverse functions. J Cell Mol Med 10: 353-363. doi:10.1111/j.1582-4934.2006.tb00404.x. PubMed: 16796804. 16796804 PMC3933126

[54] Montano MM, Ekena K, Delage-Mourroux R, Chang W, Martini P et al. (1999) An estrogen receptor-selective coregulator that potentiates the effectiveness of antiestrogens and represses the activity of estrogens. Proc Natl Acad Sci U S A 96: 6947-6952. doi:10.1073/pnas.96.12.6947. PubMed: 10359819. 10359819 PMC22022

[55] Park SE, Xu J, Frolova A, Liao L, O'Malley BW et al. (2005) Genetic deletion of the repressor of estrogen receptor activity (REA) enhances the response to estrogen in target tissues in vivo. Mol Cell Biol 25: 1989-1999. doi:10.1128/MCB.25.5.1989-1999.2005. PubMed: 15713652. 15713652 PMC549370

[56] Artal-Sanz M, Tavernarakis N (2009) Prohibitin couples diapause signalling to mitochondrial metabolism during ageing in *C.* *elegans* . Nature 461: 793-797. doi:10.1038/nature08466. PubMed: 19812672. 19812672

[57] Mao H, Wang DH, Zhou H, Yang WX (2012) Characterization and expression analysis of prohibitin in the testis of Chinese mitten crab *Eriocheir* *sinensis* . Mol Biol Rep 39: 7031-7039. doi:10.1007/s11033-012-1534-y. PubMed: 22311031. 22311031

