# Correction: Identification of a Sex-Linked SNP Marker in the Salmon Louse (*Lepeophtheirus salmonis*) Using RAD Sequencing

**DOI:** 10.1371/annotation/9a5a8f89-38b6-4ba3-aceb-65aa256f6157

**Published:** 2014-01-06

**Authors:** Stephen N. Carmichael, Michaël Bekaert, John B. Taggart, Hayden R. L. Christie, David I. Bassett, James E. Bron, Philip J. Skuce, Karim Gharbi, Rasmus Skern-Mauritzen, Armin Sturm

Multiple funding organizations and a grant to the second and last authors were incorrectly omitted from the Funding Statement. The Funding Statement should read:

"This project was financed by the Scottish Salmon Producers Organisation (SSPO) (http://www.scottishsalmon.co.uk), the University of Stirling (http://www.stir.ac.uk) and the Moredun Foundation (http://www.moredun.org.uk/charitable-wor [^]​k/the-moredun-foundation). AS and MB gratefully acknowledge support from the MASTS pooling initiative (The Marine Alliance for Science and Technology for Scotland). MASTS is funded by the Scottish Funding Council (grant reference HR09011) and contributing institutions. The funders had no role in study design, data collection and analysis, decision to publish, or preparation of the manuscript." 

